# Analysis of vitamin D status at two academic medical centers and a national reference laboratory: result patterns vary by age, gender, season, and patient location

**DOI:** 10.1186/1472-6823-13-52

**Published:** 2013-11-05

**Authors:** Jonathan R Genzen, Jennifer T Gosselin, Thomas C Wilson, Emilian Racila, Matthew D Krasowski

**Affiliations:** 1 Department of Pathology, University of Utah; ARUP Laboratories/ARUP Institute for Clinical and Experimental Pathology, Salt Lake City, UT, USA; 2 Department of Pathology and Laboratory Medicine, Weill Cornell Medical College/New York Presbyterian Hospital, New York, NY, USA; 3 Department of Psychology, Sacred Heart University, Fairfield, CT, USA; 4 Department of Pathology, University of Iowa Hospitals and Clinics, Iowa City, IA, USA

**Keywords:** Vitamin D, Vitamin D deficiency, Vitamin D toxicity, Hypervitaminosis D, Calcidiol, Ergocalciferol, Cholecalciferol, UV exposure

## Abstract

**Background:**

Testing for 25-hydroxyvitamin D [25(OH)D] has increased dramatically in recent years. The present report compares overall utilization and results for 25(OH)D orders at two academic medical centers - one in New York and one in Iowa – in order to characterize the vitamin D status of our inpatient and outpatient populations. Results are also compared to those from a national reference laboratory to determine whether patterns at these two institutions reflect those observed nationally.

**Methods:**

Retrospective data queries of 25(OH)D orders and results were conducted using the laboratory information systems at Weill Cornell Medical College / New York Presbyterian Hospital (WCMC), University of Iowa Hospitals and Clinics (UIHC), and ARUP Laboratories (ARUP). Chart review was conducted for cases with very high or low serum 25(OH)D levels in the WCMC and UIHC datasets.

**Results:**

The majority of tests were ordered on females and outpatients. Average serum 25(OH)D levels were higher in female versus male patients across most ages in the WCMC, UIHC, and ARUP datasets. As expected, average serum 25(OH)D levels were higher in outpatients than inpatients. Serum 25(OH)D levels showed seasonal periodicity, with average levels higher in summer than winter and correlating to regional UV index. Area plots demonstrated a peak of increased 25(OH)D insufficiency / deficiency in adolescent females, although overall worse 25(OH)D status was found in male versus female patients in the WCMC, UIHC, and ARUP datasets. Surprisingly, improved 25(OH)D status was observed in patients starting near age 50. Finally, chart review of WCMC and UIHC datasets revealed over-supplementation (especially of ≥ 50,000 IU weekly doses) in the rare cases of very high 25(OH)D levels. General nutritional deficiency and/or severe illness was found in most cases of severe 25(OH)D deficiency.

**Conclusions:**

25(OH)D status of patients seen by healthcare providers varies according to age, gender, season, and patient location. Improved 25(OH)D status was observed later in life, a finding that may reflect the previously described increased use of vitamin D-containing supplements in such populations. Severe vitamin D deficiency is much more common than vitamin D toxicity.

## Background

Vitamin D is a fat-soluble vitamin important for calcium absorption and bone health. Vitamin D also plays an important role in a variety of other functions, including muscle strength, cellular proliferation, differentiation, and immunomodulation to name a few (see [1] for review), and deficiency may be associated with a variety of clinical conditions and disease states [2]. Vitamin D_2_ (ergocalciferol) is obtained exclusively from dietary sources, whereas vitamin D_3_ (cholecalciferol) is obtained from dietary sources as well as the conversion of endogenous 7-dehydrocholesterol by UV-B exposure to the skin. Vitamin D_2_ and vitamin D_3_ are hydroxylated to 25-hydroxyvitamin D_2_ (25(OH)D_2_) and 25-hydroxyvitamin D_3_ (25(OH)D_3_) respectively [collectively referred to as 25(OH)D; *calcidiol*] in the liver. 25(OH)D is then hydroxylated to the biologically active hormone 1,25-dihydroxyvitamin D (1,25(OH)_2_D; *calcitriol*) in the kidney and to some extent in peripheral tissues. 25(OH)D, while an inactive precursor, is the best measurement of vitamin D nutritional status (versus 1,25(OH)_2_D or vitamin D itself) due to its longer half-life, less day to day variation, and ease of measurement in the clinically relevant concentration range [3].

Growing interest in vitamin D has led to a dramatic increase in 25(OH)D testing in recent years [4]. This surge in 25(OH)D orders has been a challenge for clinical laboratories deciding whether to handle this testing in-house or as send-outs to commercial reference laboratories. Multiple vendors now offer assays for 25(OH)D testing, which include immunochemical, chromatographic, and mass spectrometric platforms [5,6]. Numerous studies, however, have demonstrated variability of 25(OH)D results across assays [7-10]. Unfortunately, variability between assays can impact studies that examine the prevalence of vitamin D deficiency in populations, as well as clinical decisions for an individual patient [11,12].

There is actually little consensus on reference intervals used to determine what are optimal, sufficient, insufficient, deficient, and/or toxic levels of 25(OH)D [13]. For example, the Institute of Medicine (IOM) has recently defined four categories of 25(OH)D status: *risk of deficiency* (<30 nmol/L; <12 ng/mL), *risk of inadequacy* (30–49 nmol/L; 12–19 ng/mL), *sufficiency* (50–125 nmol/L; 20–50 ng/mL); and *above recommended levels* (>125 nmol/L; >50 ng/mL) [14]. Data from the National Health and Nutrition Examination Survey (NHANES) was originally analyzed to assess the vitamin D status of the United States population [15], and has more recently been reviewed with these categories in mind [14]. The Endocrine Society’s 2011 clinical practice guidelines, however, describe vitamin D *deficiency* as 25(OH)D of < 50 nmol/L (<20 ng/mL) and *insufficiency* as 52.5-72.5 nmol/L (21–29 ng/mL) [16]. Reference intervals may also vary based on assay package inserts, population-specific studies, and/or reference laboratory [13].

In the present report, we compare the ordering and result patterns of 25(OH)D testing at two large academic medical centers: one in New York City (WCMC) and one in Iowa City (UIHC). We subsequently compare 25(OH)D results, as well as the distribution of results into reference intervals by age, to those observed at a national reference laboratory (ARUP). Finally, chart review was performed for cases of vitamin D toxicity and deficiency to identify possible patterns in clinical presentation and/or causality.

## Methods

### General

This report presents data from three separate retrospective reviews of 25(OH)D tests ordered by practitioners in clinical practice. No 25(OH)D testing was ordered specifically for this report. Patients for whom 25(OH)D tests were ordered represent a mixture of healthy and ill individuals, and no screening was done to include and/or exclude patients based on patient medical history in the WCMC, UIHC, and ARUP datasets. Data were not adjusted for survival, population trends, or relative probability of visiting a healthcare provider.

### WCMC study

Two different 25(OH)D assays were available for ordering by clinicians in the inpatient and outpatient electronic health systems during the period of time investigated (October 2010 through May 2012). These were 25(OH)D by DiaSorin immunoassay (sent to ARUP Laboratories, Salt Lake City, UT) and 25(OH)D by LC-MS/MS (sent to Quest Diagnostics, Teterboro, NJ). Using a protocol approved by the Weill Cornell Medical College Institutional Review Board (WCMC IRB), the WCMC Laboratory Information System (LIS) (Millennium, Cerner Corporation, North Kansas City, MO) was queried for all vitamin D-related tests sent out over this 20 month interval. Historical ordering data (2008–2010) were reviewed to determine trends in frequency. In accordance with the WCMC IRB protocol, chart review was then conducted to determine causes of vitamin D toxicity (100 ng/mL or greater based on elevated 25(OH)D immunoassay results) or extreme deficiency [based on both 25(OH)D_2_ and 25(OH) D_3_ results < 4 ng/mL on 25(OH)D assays by LC-MS/MS].

### UIHC study

Using a protocol approved by the University of Iowa Institutional Review Board Biomedical (01) subcommittee (UI IRB), the UIHC electronic health system (EpicCare, Epic Systems Corporation, Verona, WI) was queried for all 25(OH)D tests ordered from January 2000 through October 2012. Several distinct 25(OH)D assays were utilized during this interval of time, although none simultaneously. From January 2000 to mid-July 2005, the Nichols ADVANTAGE® 25-OH Vitamin D immunoassay was performed in-house. From late-July 2005 to January 2012 specimens were sent to ARUP Laboratories (DiaSorin immunoassay). An in-house assay, Abbott Architect 25-OH Vitamin D (Abbott Laboratories, Abbott Park, IL) was used from mid-January 2012 to mid-October 2012. Starting in mid-October 2012, the laboratory moved to the Roche Elecsys Vitamin D assay (Roche Diagnostics, Basel, Switzerland) on the in-house Modular E platform. Since only a partial month of data was available for October 2012, the October 2012 data were excluded from this report. In accordance with the UI IRB protocol, chart review was performed on all patients with 25(OH)D immunoassay results of 100 ng/mL or greater, or less than 5 ng/mL. That chart review was directed at medical history and potential reasons for having elevated or severely deficient 25(OH)D serum concentrations.

### ARUP study

Using a protocol approved by the University of Utah Institutional Review Board, a de-identified list of immunoassay results for 25(OH)D (DiaSorin immunoassay) was obtained from the ARUP LIS. Exclusion criteria were any specimens potentially received from WCMC or UIHC to prevent duplication of results.

### UV index

UV index information is available in the public domain from the Climate Prediction Center, National Weather Service/National Oceanic and Atmospheric Administration website [17]. UV index is defined by the National Weather Service as “forecast of the amount of skin damaging UV radiation expected to reach the earth’s surface at the time when the sun is highest in the sky (solar noon)” [18]. The daily “Issued UV Index” is used throughout this paper and incorporates cloud information (as opposed to “clear sky UV index”). Issued UV index was used from the weather station at the John F. Kennedy International Airport (~ 40.64°N latitude) in New York (near WCMC ~ 40.77°N latitude) and the weather station at the Des Moines International Airport (~ 41.53°N latitude) in Iowa (near UIHC ~41.66°N latitude).

### Limit of quantitation and exclusions

Assays for 25(OH)D have a lower limit of quantitation. Results below this are typically reported with a “less than” (<) symbol. For the duration of time evaluated in this study, the following were the lower limits of quantitation evident in results of our queries from 2000 through 2012: Nichols Advantage (< 7 ng/mL), ARUP (< 4 ng/mL or < 7 ng/mL), Abbott (< 4 ng/mL or < 7 ng/mL), Roche (< 5 ng/mL). Analysis of ordering patterns and frequency was not impacted by these “less than” results and includes all available data. Area plot analysis (see below) also includes all “less than” results, as they could be fit within the same interval category (*ex*. 0–10 ng/mL). For analysis of 25(OH)D immunoassay results by sex, age, and month, however, “less than” results were not included, as an actual value was unknown. For WCMC, 59 results (~0.1% of total results; 40 female, 19 male) were excluded from analysis of 25(OH)D averages due to “less than” results. For UIHC, 378 results (~0.6% of total results; 235 female, 143 male) were excluded due to “less than” results. For ARUP, ~0.09% of total results (64.4% female, 35.6% male) were excluded due to “less than” results. The difference in the relative percent of excluded results from UIHC (versus WCMC or ARUP) is likely due to the much longer period of data available for analysis from UIHC and corresponding improvements in 25(OH)D immunoassay performance and sensitivity over time. An additional 30 “results” (20 female, 10 male) were excluded from analysis at WCMC, as text comments instead of numeric results were retrieved from the de-identified LIS queries.

For the LC-MS/MS total 25(OH)D and fractionated 25(OH)D_2_ and 25(OH)D_3_ result analysis from the WCMC dataset, exclusion of values below the lower limit of quantitation (< 4 ng/mL; n = 6,445 for 25(OH)D_2_ and n = 75 for 25(OH)D_3_) would lead to a marked over-estimation of average 25(OH)D_2_ concentrations. For these figures, “less than” results were therefore tabulated as “zeros” for the purpose of graphical display and average 25(OH)D_2_ and 25(OH)D_3_ concentration by age. Area plot analysis of LC-MS/MS data includes all “less than” results, as they could be fit within the same interval category.

Finally, it should be noted that repeat testing on the same patient (pseudoreplication) could not be avoided in the WCMC, UIHC, and ARUP datasets, as patient-level exclusion was not possible on our IRB-approved de-identified result queries. A separate analysis of WCMC 25(OH)D ordering, however, revealed the following distribution of 25(OH)D “orders per patient” in that interval of time studied (20 months): 1 order, 69.3%; 2 orders, 19.5%; 3 orders, 6.2%; 4 orders, 2.4%; 5 orders, 1.2%; 6 orders, 0.6%; 7 orders, 0.3%; 8 orders, 0.2%; 9 orders, 0.1%; ≥ 10 orders, 0.2%.

### Data analysis

Microsoft Excel 2007 and SigmaPlot 11 (Systat Software, Inc, Chicago, IL) were used to analyze and visualize data by age, sex, order month, and patient location. Patient age was rounded to the nearest year to facilitate binning and comparison. Alternate (quantitative) method comparison between immunoassay and LC-MS/MS (including bias, correlation coefficient, and Deming regression) was conducted in EP Evaluator 9 (Data Innovations, South Burlington, VT) and plotted in SigmaPlot 11. Cross-tabulation in SPSS 18 (PASW; IBM, Armonk, NY) was used to calculate the proportion of results by age that fall into specific reference intervals. These results were used to generate area plots in SigmaPlot, which display stacked, proportionate areas such that the sum at age is equal to 100% (see corresponding legend for a description). Data throughout the manuscript are presented as mean ± standard deviation (SD) unless otherwise indicated. Since results demonstrated a non-normal 25(0H)D distribution of scores, statistical significance was evaluated using the Mann Whitney rank sum test (with alpha set at 0.05 for the threshold of significance).

## Results

### 25(OH)D by immunoassay

In the 20 months of testing analyzed at WCMC, 57,433 clinician orders for 25(OH)D by immunoassay and 8,439 orders for 25(OH)D by LC-MS/MS were identified. As 25(OH)D results can differ by methodology, WCMC LC-MS/MS orders were not included in initial ordering and result analysis, but are analyzed separately and in the chart review of deficiency cases. In the 153 months analyzed at UIHC, 60,237 orders for 25(OH)D by immunoassay were identified. Total number of immunoassay results reviewed from WCMC, UIHC, and ARUP are included in Table 1. Table 2 provides summary statistics by platform for 25(OH)D immunoassay testing at UIHC.

**Table 1:** **25**(**OH**)**D orders and immunoassay results by institution**, **patient location**, **and sex**

				***Average 25(0H)D Results ± SD (ng/mL)***			***p value***
	**Patient location**	**# Orders females**	**# Orders males**	**Female (F)**	**Male (M)**	**All**	***F vs M***
**WCMC**^**a**^	Private Ambulatory	28911	12766	30.5 ± 13.3	28.4 ± 12.7	29.8 ± 13.2	p < 0.001
	Hospital Outpatient Clinics	9046	3357	27.2 ± 12.4	24.9 ± 11.3	26.6 ± 12.1	p < 0.001
	Inpatient	1605	999	25.8 ± 19.2	21.9 ± 12.1	24.3 ± 16.9	p < 0.001
**UIHC**	Outpatient	37684	17153	30.5 ± 14.6	29.8 ± 14.3	30.2 ± 14.5	p < 0.001
	Dialysis^b^	67	54	26.1 ± 13.0	32.6 ± 16.3	29.0 ± 14.8	p = 0.031
	Emergency Department	51	22	28.8 ± 17.5	24.8 ± 10.5	27.6 ± 15.8	p = 0.574
	Inpatient	2930	1923	27.3 ± 16.0	25.7 ± 18.3	26.7 ± 17.0	p < 0.001
	Inpatient ICU^c^	167	186	22.6 ± 13.4	24.8 ± 14.0	23.8 ± 13.7	p = 0.119
**ARUP**	Unknown^d^	414640	184208	29.4 ± 13.8	28.1 ± 13.0	29.0 ± 13.6	p < 0.001

^a^A total of 749 WCMC results (437 female, 312 male) were omitted from this table due to uncertain designation of inpatient versus outpatient status. WCMC ED category not included as there were only 14 females and 2 males.

^b^All UIHC dialysis patients were considered outpatients for the purpose of this analysis.

^c^UIHC inpatient ICU includes data from cardiovascular ICU, medical ICU, neonatal ICU, and pediatric ICU.

^d^Inpatient versus outpatient status was not identifiable. Specimens received from WCMC and UIHC were excluded from ARUP result analysis.

**Table 2:** **25**(**OH**)**D immunoassay results by assay platform** – **UIHC dataset**

					***Average 25(0H)D Results ± SD (ng/mL)***		
	**Assay**	**Dates used**	**# Orders females**	**# Orders males**	**Female (F)**	**Male (M)**	**All**
**UIHC**	Nichols ADVANTAGE® (in-house)	Jan 2000 - Jul 2005	1689	998	27.4 ± 16.6	24.9 ± 18.0	26.5 ± 17.2
	DiaSorin Immunoassay (send-out)	Jul 2005 -Jan 20012	32602	15092	30.6 ± 14.7	30.0 ± 15.0	30.4 ± 14.8
	Abbott Architect (in-house)	Jan 2012 -Oct 2012	6608	3248	29.4 ± 14.2	27.6 ± 12.2	28.8 ± 13.6

**Figure 1:**
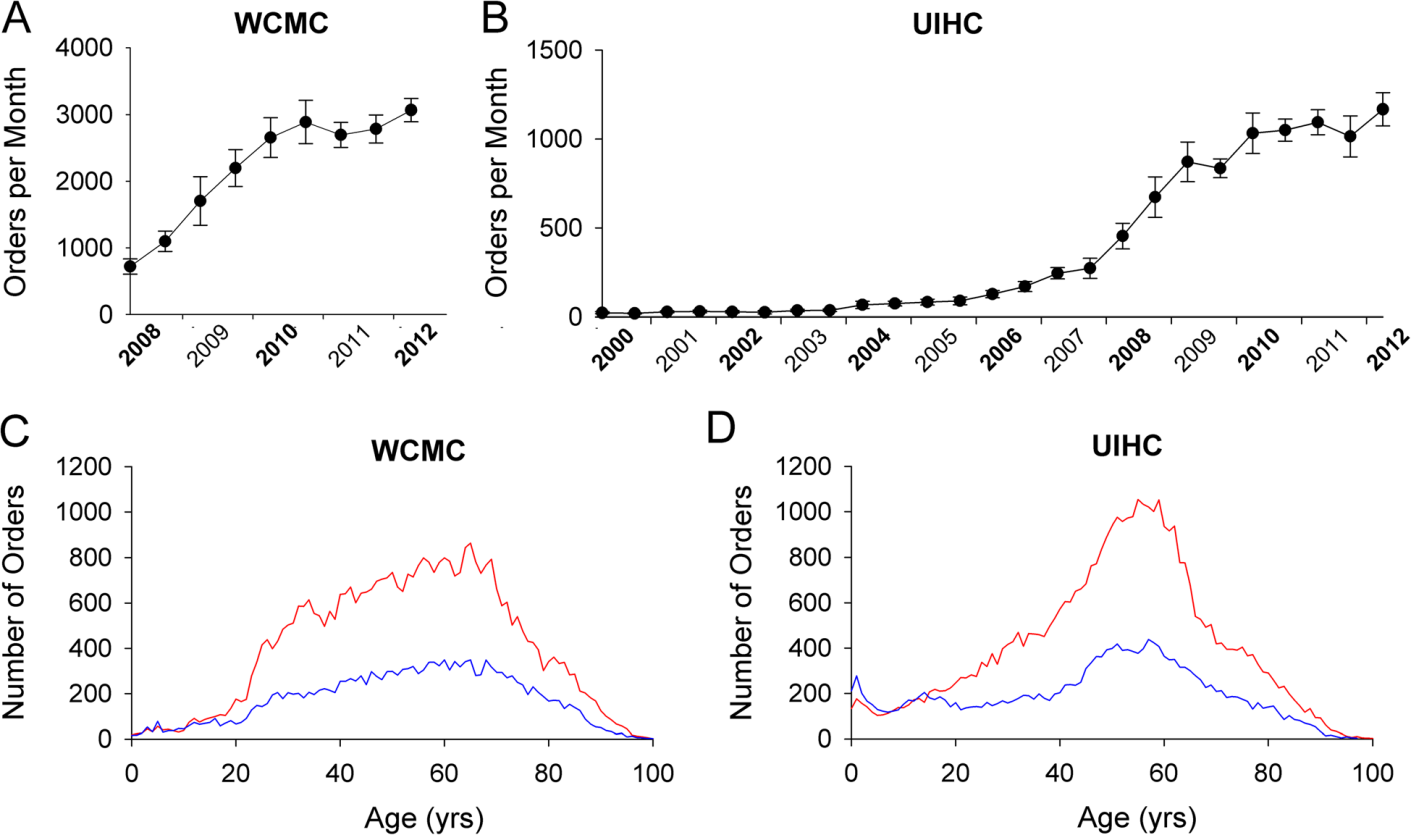
**25(OH)D immunoassay - orders by institution, age, and sex. A**, **B**. Average number of 25(OH)D orders per month (in six month bins) at WCMC **(A)** and UIHC **(B)**. Error bars are ± SD. **C**,**D**. Total number of orders by patient age and sex (female = red lines; male = blue lines) at WCMC (**C**; 20 months analyzed) and UIHC (**D**; 153 months analyzed). Data are binned to 1 yr age intervals.

The vast majority of tests were ordered on outpatients (versus inpatients) at both WCMC (95.4%) and UIHC (91.2%) (Table 1). The total number of monthly 25(OH)D orders increased dramatically over the interval of time studied at both WCMC (Figure 1A) and UIHC (Figure 1B). This increase was most prominent between 2008 and 2010 at both institutions (ex. 2008 to 2009 annual growth rates were 115% at WCMC and 52% at UIHC). The majority of tests were ordered on female patients, with similar percentages observed across datasets (WCMC 69.8%, UIHC 67.9%; see Table 1). The number of 25(OH)D orders in female and male patients at WCMC (Figure 1C) and UIHC (Figure 1D) are shown by patient age. At both institutions, the predominance of orders in female (versus male) patients begins at approximately age 20 and continues thereafter. At WCMC, the number of test orders in both female and male patients increased steadily between ages of approximately 20 through 70 years old before decreasing (Figure 1C). At UIHC, the increase in test orders was more pronounced between the ages of approximately 40 through 60 years old before decreasing (Figure 1D). A greater number of orders for pediatric and adolescent patients were observed at UIHC than WCMC (Figure 1C,D), although this may also represent differing patient populations, as New York Presbyterian Hospital’s largest children’s hospital is not on the WCMC campus (therefore, corresponding specimens/results are not included in the WCMC dataset).

**Figure 2:**
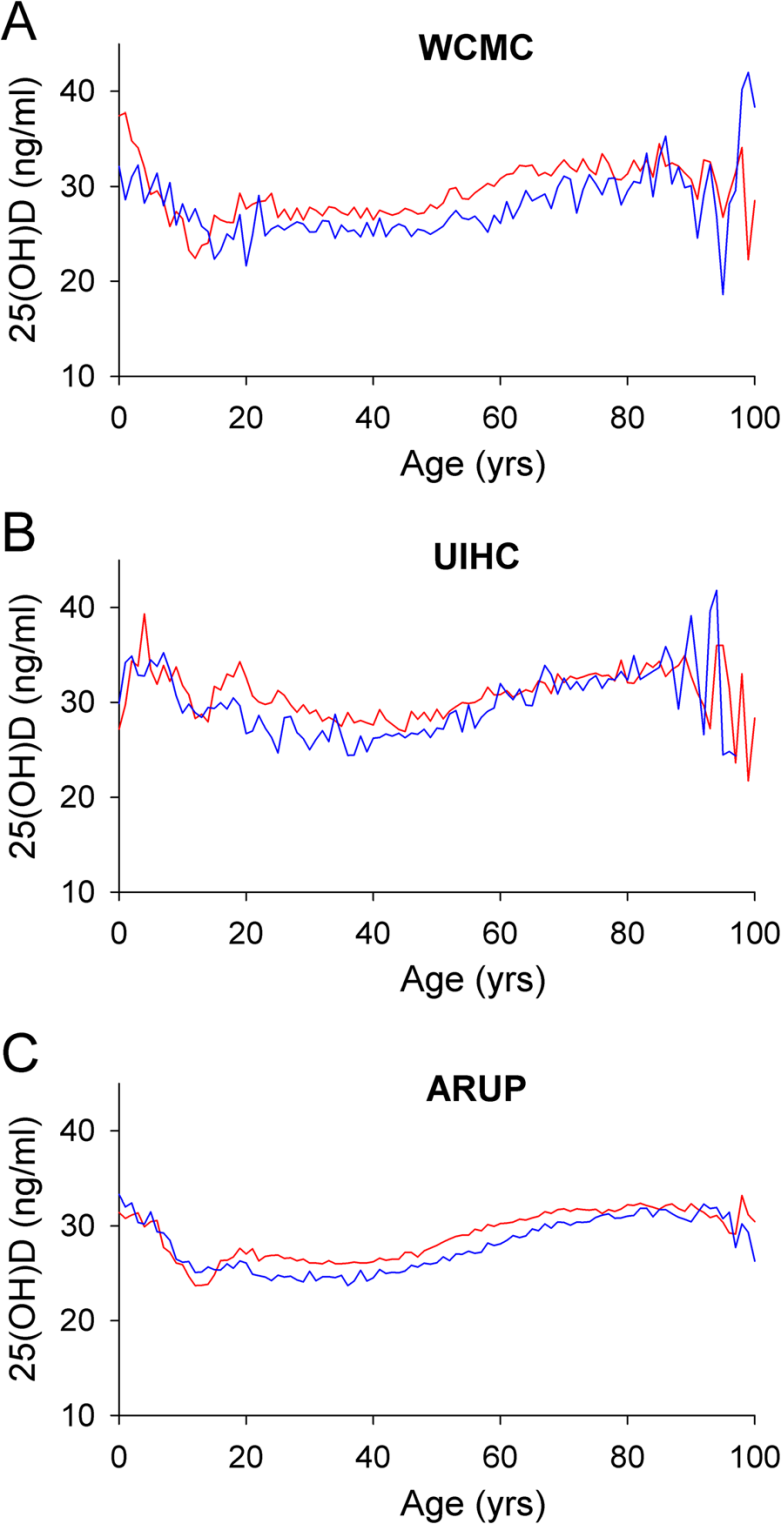
**25(OH)D immunoassay – average concentration by age and sex – results for all ages.** Average 25(OH)D results at WCMC **(A)**, UIHC **(B)**, and ARUP **(C)** by age and sex (female = red lines; male = blue lines). Variability at young and old ages (particularly **A** and **B**) may be due to comparatively small n-values (see Figure 1C,D).

Figure 2 shows average 25(OH)D results by age and gender for WCMC (Figure 2A), UIHC (Figure 2B), and ARUP (Figure 2C) datasets. Each set shows a general decline in average 25(OH)D level in adolescence, as well as a relative stability of average 25(OH)D level between the ages of approximately 20 to 50 years old. In each of the datasets, average 25(OH)D levels increased after age 50 in both men and women. It is important to note that population sizes for pediatric and geriatric patients in the WCMC and UIHC datasets are relatively small (see Figure 1C,D), a factor that likely contributes to the variability in results at very young and very old ages (Figure 2A,B). This variability is therefore less pronounced in the much larger ARUP dataset (Figure 2C).

**Figure 3:**
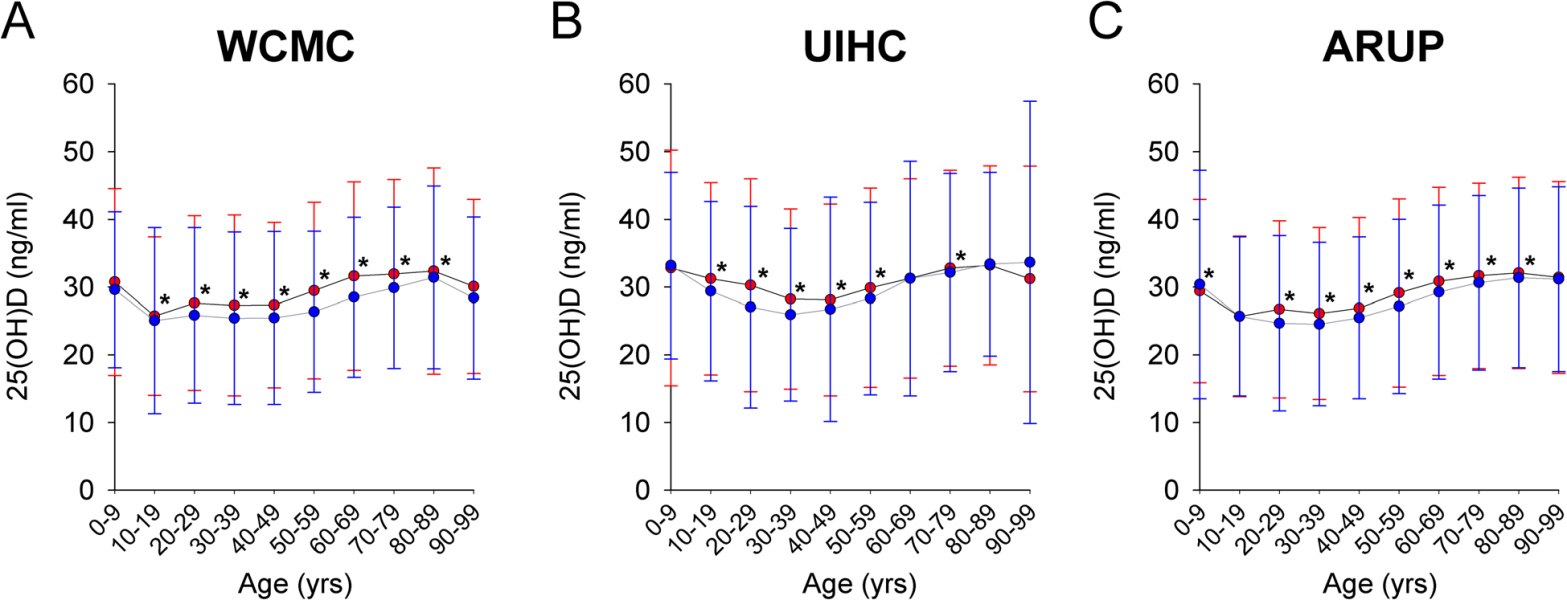
**25(OH)D immunoassay – average concentration by age and sex – results by decade of life. A**,**B**. Average 25(OH)D results at WCMC **(A)**, UIHC **(B)**, and ARUP **(C)** by age (in 10 year bins) and sex [female = red circles; male = blue circles]. Error bars are ± SD. * p < 0.05.

Average 25(OH)D results by decade of life (with SD) is presented in Figure 3. While the SD of results within age groups (by gender) is large with overlapping error bars, statistically significant differences were found between female and male patients at most ages. At WCMC (Figure 3A), UIHC (Figure 3B), and ARUP (Figure 3C), average 25(OH)D results were significantly higher in females than males at most ages (notable exception ARUP 0–9 yr olds, Figure 3C), although this trend was more pronounced at WCMC and ARUP versus UIHC.

Table 1 presents average 25(OH)D results (including all ages) for female and male patients by location. The only locations where average 25(OH)D results were statistically higher in men than women had relatively small population sizes (<200 per group). At WCMC, both outpatient populations (private ambulatory and hospital-owned clinics) showed higher 25(OH)D results than inpatients for both women and men (p < 0.001). Both female and male 25(OH)D levels were significantly lower in patients at the hospital-owned clinics versus private ambulatory clinics (p < 0.001), a factor that may be related to different patient populations served and/or ordering practices at these two categories of clinics. At UIHC, 25(OH)D results were also higher in outpatients than inpatients for both genders (p < 0.001). 25(OH)D results were significantly lower in female ICU inpatients than female non-ICU inpatients (p < 0.001), although this was not true when comparing male results between ICU and non-ICU inpatients (p = 0.648).

**Figure 4:**
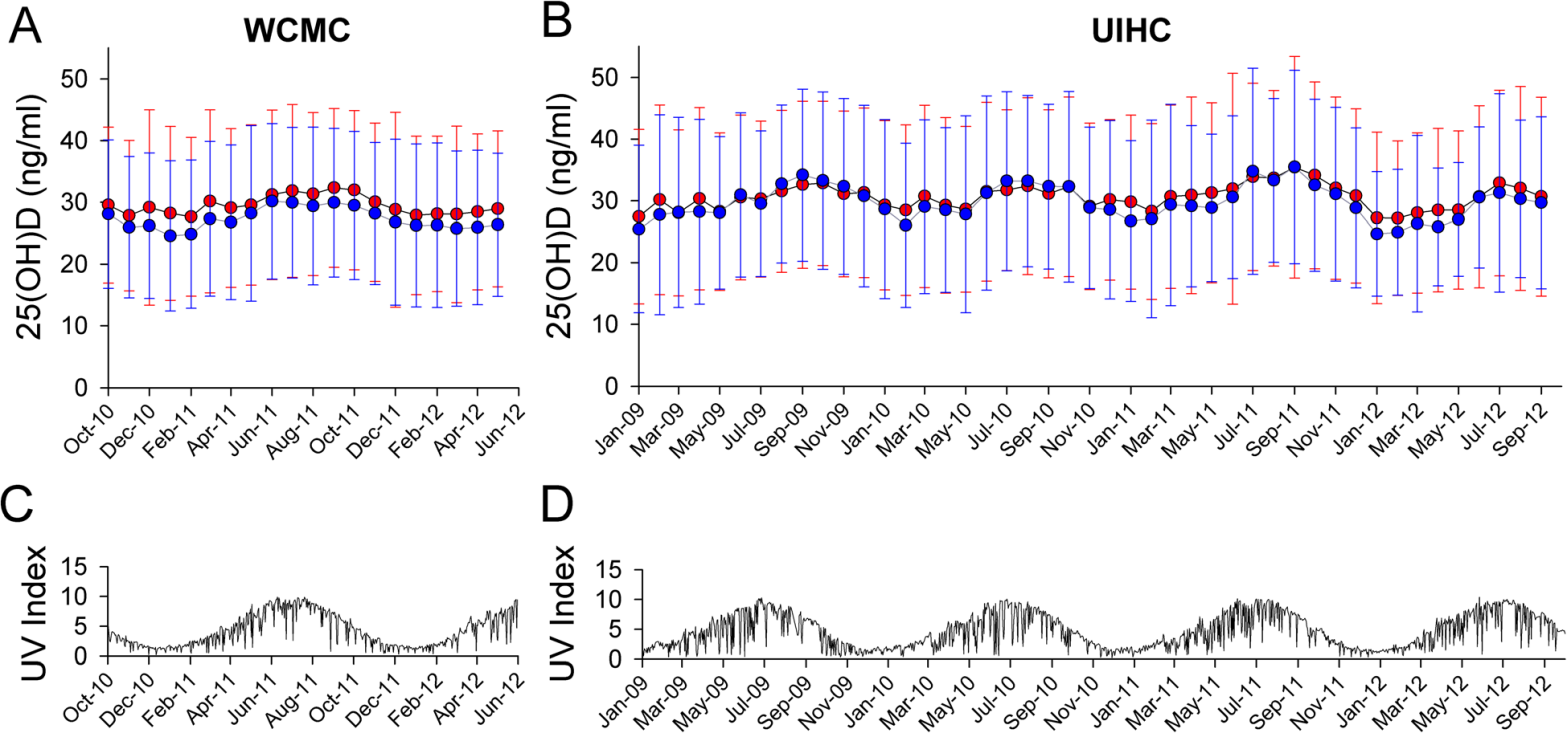
**25(OH)D immunoassay – average concentration by order month with regional UV index. A**,**B**. Average 25(OH)D results at WCMC **(A)** and UIHC **(B)** by sex [female = red circles; male = blue circles] and order month. Error bars are ± SD. **C**,**D**. Daily issued UV Index for New York City, NY **(C)** and Des Moines, IA **(D)** (see Methods).

As it is known that sunlight (specifically UV-B) exposure promotes peripheral conversion of 7-dehydrocholesterol to previtamin D, we analyzed average 25(OH)D results for males and female patients by order month (Figure 4A,B) and compared this to reported regional UV Index data from the National Weather Service (see Methods; Figure 4C,D) recorded at monitoring locations nearest to the respective medical centers. As expected, 25(OH)D results were higher in summer than winter months in both male and female patients with patterns following the seasonal trends as evident in UV Index periodicity.

**Figure 5:**
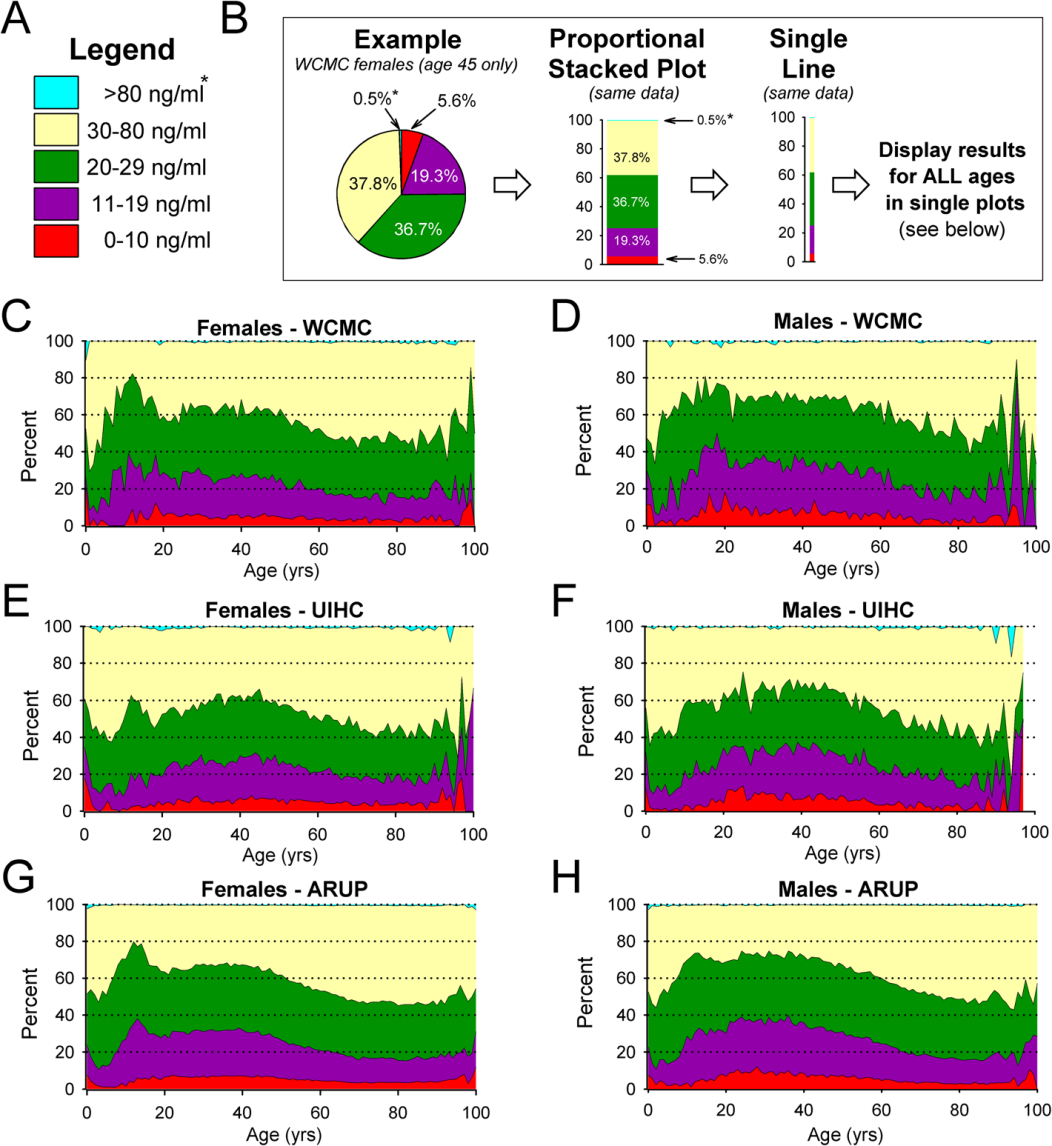
**25(OH)D immunoassay – results by reference intervals. A**. Figure legend showing the colors representing reference intervals used in **B** through **H**. An asterisk (*) is indicated next to the >80 ng/mL intervals (blue) in **A** and **B**, as this interval often contains too few specimens to be easily visible at this magnification (also evident in **C** through **H**). **B**. Presentation of population data as area plots. For any given group (for example, WCMC female patients age 45 shown here) the percent of patients that fall into specific reference intervals can be represented as a pie chart (**B**, left example). To the right of the pie chart is a “proportional stacked plot” (**B**, middle) that shows the same data, but now stacked by color-coded reference intervals (low to high) such that the sum of all areas equals 100%. These data can also be presented as a narrower stacked color-coded “single line” (**B**, right). Graphing “single line” area plots for all ages (aligned by increasing age) produce the area plots shown below. **C-H**. Area plots show the reference interval distributions for *all* ages (0–100 yrs; 1 yr bins) in females **(C,E,G)** and males **(D, F, H)** at WCMC **(C,D)**, UIHC **(E,F)** and ARUP **(G,H)**. The UIHC dataset does not include any males greater than 97 years old **(F)**. Overlying reference lines (dotted) have been added at 20% intervals to all area plots **(C-H)** to facilitate comparison across graphs.

To view the distribution of all patient results within specific reference intervals, proportional stacked area plots were generated for WCMC (Figure 5C,D), UIHC (Figure 5E,F), and ARUP (Figure 5G,H) and divided into female patients (left column) and male patients (right column). The reference intervals for ARUP (instead of IOM) were chosen as a starting point for area plot generation, since the majority of specimens in this report were actually tested at ARUP. The < 20 ng/mL interval, however, was subdivided into two separate intervals (0–10 ng/mL and 11–19 ng/mL) to improve our ability to view deficiency in these graphs. Since cases of toxicity were rare, data from the >150 ng/mL “*possible toxicity*” interval used by ARUP were merged into the >80 ng/mL interval as they were too small to be visible when plotted on the graphs. Even the >80 ng/mL interval is difficult to visualize in Figure 5 (blue shading; not always present at a given age). A color coded legend of the intervals displayed is indicated in Figure 5A and applies to all other area plots (Figure 5C-H). A description of area plot generation is also provided in the Methods section and is illustrated in Figure 5B (and figure legend) to assist the reader.

A peak of sub-optimal 25(OH)D status is again evident in female adolescents (see Figure 5G ARUP data), although in general 25(OH)D status is lower in male patients in the WCMC, UIHC, and ARUP datasets (as suggested by upper border of magenta interval, Figure 5C-H). A relative stability of 25(OH)D status is again evident between the ages of approximately 20–50 years old in WCMC (Figure 5C,D), UIHC (Figure 5E,F), and ARUP (Figure 5G,H) datasets. An increase in optimal 25(OH)D levels (yellow shading) was observed after approximately age 50 in the WCMC, UIHC, and ARUP datasets.

### 25(OH)D by LC-MS/MS

**Figure 6:**
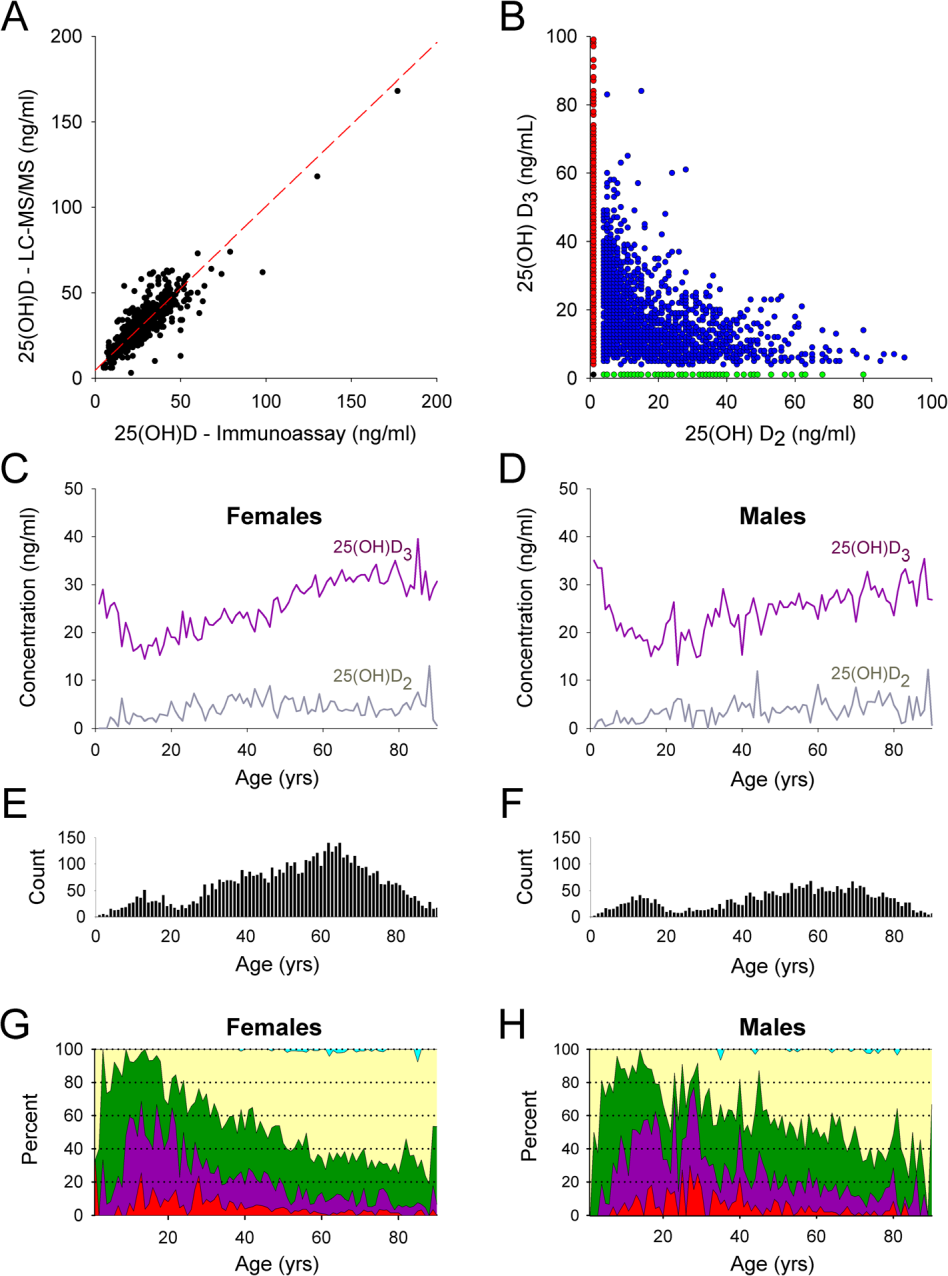
**25**(**OH**)**D by LC**-**MS**/**MS** - **WCMC dataset. A**. 25(OH)D immunoassay results versus LC-MS/MS total 25(OH)D results in cases where both tests were ordered by clinicians on the same specimen. Deming regression (red dashed line) shows a slope of 0.958 and a Y-intercept of 4.7 ng/ml. **B**. Corresponding fractionated 25(OH)D_2_ and 25(OH)D_3_ results from specimens ordered for 25(OH)D testing by LC-MS/MS from WCMC. See Results section for n-values. Many data points are obscured due to overlapping position. Both X- and Y-axes are cropped at 100 ng/ml to improve visibility. Red dots (many overlapping) = unquantifiable 25(OH)D_2_, quantifiable 25(OH)D_3_. Green dots = quantifiable 25(OH)D_2_, unquantifiable 25(OH)D_3_. Black dot (overlapping) = unquantifiable 25(OH)D_2_ and unquantifiable 25(OH)D_3_. Blue dots = quantifiable 25(OH)D_2_ and quantifiable 25(OH)D_3_. **C**,**D**. Average 25(OH)D_2_ (gray line) and average 25(OH)D_3_ (magenta line) results age and sex (**C**, females; **D**, males) at WCMC. Variability may be due to comparatively small n-values as shown by specimen counts in each 1 yr bin (**E**, females; **F**, males). **G**,**H**. Area plots show the reference interval distributions for all ages (1–90 yrs; 1 yr bins) in females **(G)** and males **(H)** at WCMC. Figure legend (color) for area plots is identical to Figure 5A.

The WCMC inpatient and outpatient electronic health systems permitted clinicians to electronically order 25(OH)D by immunoassay and/or 25(OH)D by LC-MS/MS, as described in the Methods section. Given the availability of both assays, in our dataset review we identified 443 cases in which both immunoassay and LC-MS/MS were ordered by clinicians on the same specimen. Analysis of these paired results (Figure 6A), revealed a correlation coefficient (R) of 0.85 and an overall bias (LC-MS/MS to immunoassay) of 3.5 ng/ml (11.5%). As the LC-MS/MS assays were reported with three components - total 25(OH)D and fractionated 25(OH)D_2_ and 25(OH)D_3_ – it was possible to plot 25(OH)D_2_ versus 25(OH)D_3_ for specimens in which LC-MS/MS was ordered (n = 8,439). This analysis (Figure 6B) revealed that in the majority of specimens (76.4%; n = 6,445; overlapping red dots), 25(OH)D_2_ was not quantifiable while 25(OH)D_3_ was quantifiable. In a small minority of specimens (0.89%; n = 75; green dots), 25(OH)D_2_ was quantifiable while 25(OH)D_3_ was not quantifiable. In a smaller minority of specimens (0.20%; n = 17; overlapping black dot), neither were quantifiable. In the remaining specimens (22.5%; n = 1,902; blue dots), both 25(OH)D_2_ and 25(OH)D_3_ were quantifiable.

The LS-MS/MS dataset also allowed us to plot 25(OH)D_2_ and 25(OH)D_3_ by age for female (Figure 6C) and male (Figure 6D) patients. While there is significant scatter in the data due to the relatively small number of specimens for patients at each age (Figure 6E, females; Figure 6F, males; 1 yr bins), the overall trend in 25(OH)D_3_ results is similar to the 25(OH)D immunoassay results observed in Figure 2A-C, supporting a hypothesis that the increase in optimal 25(OH)D concentrations observed in later adulthood (see Figures 2, 3, and 5) are primarily due to increased concentrations of serum 25(OH)D_3_. Reference range area plots were also generated for total 25(OH)D by LC-MS/MS at WCMC (Figure 6G, females; Figure 6H, males). While increased scatter is again noted due to the relatively small number of specimens, the overall pattern is relatively consistent with that observed in 25(OH)D immunoassay results (Figure 5).

### Chart review – elevated 25(OH)D

Chart review was performed on all UIHC and WCMC patients who had 25(OH)D levels of 100 ng/mL or greater (see Table 3). At UIHC, over the nearly 13 years of results reviewed, only 27 patients (~0.05%) had 25(OH)D levels greater than 150 ng/mL. An additional 82 patients (~0.14%) had 25(OH)D levels between 100 and 150 ng/mL. The highest 25(OH)D level at UIHC (851 ng/mL) was seen in a patient with X-linked hypophosphatemia. There were only 3 additional patients with a 25(OH)D level of 300 ng/mL or higher. For patients with 25(OH)D of 126 ng/mL or greater in the UIHC dataset, 27.5% were observed in patients taking a 50,000 unit vitamin D_2_ prescription more often than once weekly. An additional 31.4% were attributed to patients taking very high doses of over-the-counter vitamin D supplements outside of physician recommendations. Interestingly, only 3 patients out of the 109 total who had 25(OH)D of 100 ng/mL or greater had hypercalcemia (defined as above upper limit of age-specific reference range for total and/or ionized calcium). Only one of these patients was hospitalized for management of hypercalcemia - a 70 year old female with dementia and acute renal failure in addition to hypercalcemia with a 25(OH)D level of 194 ng/mL. There were no cases of vitamin D-based rodenticide poisoning in the UIHC dataset.

**Table 3:** **Chart review of patients with 25**(**OH**)**D** ≥ **100 ng**/**mL**

				**Number of patients**				
	**25(OH)D level (ng/ml)**	**Number of patients**	**Average age ± SD (yrs)**	**Number of females**	**Number of pediatric patients**^**a**^	**Suspected cause of elevated 25(OH)D**		**Hypercalcemia at time of 25(OH)D measurement**
							**Over-the-counter vitamin D supplements**	
UIHC^b^	> 200	11	43.9 ± 18.3	5 (45.4%)	1 (9.1%)	4 (36.3%)	4 (36.3%)	0
	151 – 200	16	58.0 ± 19.6	10 (62.5%)	2 (12.5%)	4 (25.0%)	6 (37.5%)	2 (12.5%)
	126 – 150	24	53.1 ± 17.4	18 (75.0%)	0	6 (25.0%)	6 (25.0%)	1 (4.2%)
	100 – 125	58	49.5 ± 20.0	38 (65.5%)	2 (3.4%)	6 (10.3%)	4 (6.9%)	0
WCMC	> 200	5	58.8 ± 21.5	4 (80.0%)	0	1 (20%)	3 (60%)	4 (80%)
	151 – 200	10	46.0 ± 14.9	7 (70.0%)	0	3 (30%)	4 (40%)^c^	0
	126 – 150	6	58.8 ± 12.1	6 (100.0%)	1 (16.7%)	1 (16.7%)	0^d^	0
	100 – 125	29	57.0 ± 16.8	25 (86.2%)	0	4 (13.8%)	10 (34.5%)	0

^a^Age < 18 years old.

^b^Including repeat measurements on patients, there were 16, 22, 27, and 75 total measurements in the > 100 ng/mL, 151–200 ng/mL, 1260–150 ng/mL, and 100–125 ng/mL categories, respectively.

^c^One additional patient received monthly 50,000 IU Vitamin D doses, not listed as given by prescription.

^d^One additional patient received “Vitamin B12 and D” injections (no doses listed), not listed as given by provider.

In the UIHC review there were only 3 pediatric patients (17 years or younger) who had 25(OH)D of 126 ng/mL or greater. The pediatric patients were 23 months old (182 ng/mL), 4 years old (292 ng/mL) and 17 years old (146 ng/mL). The elevated 25(OH)D in the first two patients were attributed to high prescription vitamin D doses which were adjusted once the elevated 25(OH)D serum concentration was known.

At WCMC, over the 20 months of results reviewed, only 15 patients (~0.03%) had 25(OH)D levels > 150 ng/mL, with an additional 35 (~0.06%) having a measurement between 100 and 150 ng/mL. Vitamin D supplementation was specifically mentioned in 29 of these 50 cases, with actual and/or estimated doses documented in 24 of these cases. In 10 cases “vitamin D deficiency” was mentioned in prior clinic notes, suggesting that the elevated 25(OH)D result was in response to therapy. Four of the 50 WCMC patients with 25(OH)D levels of 100 ng/mL or greater had hypercalcemia, and each of these four had 25(OH)D levels > 150 ng/mL. As with UIHC, there were no cases of vitamin D-based rodenticide poisoning in the WCMC dataset.

The highest 25(OH)D level at WCMC (409 ng/mL) was in a patient hospitalized for symptoms associated with severe hypercalcemia. This patient subsequently disclosed to clinicians that she had been consuming massive amounts of over-the counter vitamin D and calcium-containing supplements. Only one other patient had a 25(OH)D level of 300 ng/mL or greater. That patient (an elderly male with dementia, chronic renal insufficiency, and recurrent Staghorn calculi) had been diagnosed one year earlier by his clinician with “vitamin D deficiency” due a 25(OH)D level of 24 ng/mL. It was unclear what the prescribed and/or consumed amount of vitamin D supplementation was, nor was it evident whether the history of renal calculi pre-dated the vitamin D supplementation. At WCMC, there was only one pediatric patient with a 25(OH)D value of 100 ng/mL or greater, a 4 month old diagnosed with osteogenesis imperfecta (25(OH)D result of 130 ng/mL) receiving vitamin D supplementation.

In 12 of the 50 WCMC patients identified with 25(OH)D levels of 100 ng/mL or greater, bone disease (osteoporosis, osteopenia, scoliosis, and/or fracture) was documented by the clinicians. Four of the 50 WCMC cases showed vitamin D supplementation in the context of Crohn’s disease, ulcerative colitis, and/or inflammatory bowel disease. Five of the 50 WCMC cases showed vitamin D supplementation in the context of multiple sclerosis.

### Chart review – decreased 25(OH)D

Chart review was also conducted to investigate possible causes for severe 25(OH)D deficiency in UIHC and WCMC patients (Table 4). For the UIHC dataset, chart review was conducted for cases of “severe vitamin D deficiency” with total 25(OH)D < 5 ng/mL. This occurred in 185 patients over a 13 year period. A likely primary cause of severe vitamin D deficiency could be ascertained from chart review in 134 out of 185 patients (72.4%). The most common suspected causes were severe dysfunction of the liver and/or biliary tract (n = 26), lipid malabsorption syndrome (n = 25, examples included cystic fibrosis, abetalipoproteinemia, celiac disease, and inflammatory bowel disease), and morbid obesity/post-gastric bypass surgery (n = 23). Only 54 of 185 (29.2%) patients had a total calcium serum concentration below age-appropriate reference range at time of 25(OH)D measurement. Forty patients were not taking vitamin D supplements prior to 25(OH)D measurements.

**Table 4:** **Clinical characteristics for patients with extremely low 25**(**OH**)**D results**^**a**^

	**Number of patients**	
**Clinical characteristics**	**UIHC**	**WCMC**
*Decreased production or intake of vitamin D*		
Severe malnutrition	6	-^b^
Skin damage (e.g., burns)	0	0
*Malabsorption of vitamin D or deficient 25*-*hydroxylation*		
Liver failure and/or biliary tract dysfunction	26	1
Other disorder with lipid malabsorption	25	0
*Increased loss of 25*(*OH*)*D*		
Nephrotic syndrome	5	1
Renal failure	23	2
*Increased catabolism or 1α*-*hydroxylation of 25*(*OH*)*D*		
Liver-enzyme inducing medications	7	0
Hyperthyroidism	0	0
Granulomatous disease	4	0
*Other conditions possibly linked to 25*(*OH*)*D deficiency*		
Morbid obesity and/or status post bariatric surgery	23	4
Systemic lupus erythematosus without lupus nephritis	8	0
Systemic lupus erythematosus with lupus nephritis	4	0
*Unknown*/*Other*	51	3
Perinatally acquired HIV; now in adolescence or adulthood	0	2
Primary hyperparathyroidism	3	0

^a^Thresholds for Deficiency Chart review were: UIHC, 25(OH)D immunoassay results of <5 ng/mL; WCMC, 25(OH)D LC-MS/MS results where both D_2_ and D_3_ were <4 ng/mL.

^b^At WCMC, six of the cases noted signs of malnourishment and/or documented poor appetite. These cases are categorized, however, under the primary causative Clinical Characteristics to eliminate duplication.

For the WCMC dataset, chart review was conducted for cases of “severe deficiency” where 25(OH)D_2_ and 25(OH)D_3_ results by LC-MS/MS were both <4 ng/mL. Of 8,439 orders for 25(OH)D by LC-MS/MS, 14 distinct patients (0.17%) met this criteria. One of these 14 patients, however, had a separate 25(OH)D result by immunoassay reported on the same specimen with discordant results of 21 ng/mL. As no clinical history was available for this patient, it was excluded from further analysis due to possible mix-up and/or analytical error. Of the remaining 13 patients (see Table 4), three had a history of prior bariatric surgery (one with complications), one presented for a pre-operative evaluation for bariatric surgery, three had renal failure (two were status post renal transplant while the other had nephrotic syndrome), one had liver failure (pre-transplant evaluation), and three did not have a clear documented cause. It should be noted that in six of the cases, signs of malnourishment and/or poor appetite were documented by the clinicians. Interestingly, two patients had a history of perinatally acquired HIV but were now in late adolescence or adulthood, although it should be emphasized that poor dietary habits were noted for one of these two cases as well. Chart review revealed documentation of subsequent vitamin D supplementation in 8 of the 13 cases reviewed.

## Discussion

A number of studies have examined vitamin D status in large populations. For example, comprehensive analyses of NHANES data have previously been conducted [14,15,19]. A few of the findings of NHANES differ, however, from observations described here for the WCMC, UIHC, and ARUP clinical datasets. For example, the NHANES studies indicated that 25(OH)D concentrations are, in general, higher in males than females [15,19]. A second observation of the published NHANES data is that children tended to have higher 25(OH)D concentrations than adults [15]. While we observed a general pattern of more optimal 25(OH)D status in childhood in all datasets analyzed, an improvement in 25(OH)D status later in adulthood was observed at WCMC, UIHC, and ARUP, demonstrating that 25(OH)D status changes throughout life. For the WCMC dataset, we were able to demonstrate this pattern using both immunoassay and LC-MS/MS 25(OH)D results.

It is likely that consumption of vitamin D fortified foods, as well as UV exposure during childhood [20,21] support 25(OH)D status during youth. Studies of the Canadian Health Measures Surveys data have been able to demonstrate that vitamin D supplementation contributes to improved 25(OH)D status [22], as was frequent milk consumption and white racial background among other factors [23]. It should be noted that American female adolescents report consuming less milk than male adolescents [24], and in general, girls (during childhood and adolescence) have been reported to spend less time outdoors than boys [21]. These factors may contribute to the patterns observed for female adolescents throughout this report.

We suspect that increased use of vitamin D-containing supplements (specifically vitamin D_3_) during later adulthood contributes to the improved 25(OH)D status observed at those ages [25]. Elevation of 25(OH)D_3_ levels as measured by LC-MS/MS (Figure 6C,D) supports this hypothesis. Increased use of supplemental vitamin D in women versus men is also likely to contribute to the more optimal 25(OH)D status in women seen in our report [25].

It should be noted that another large study of approximately 158,000 individuals who had 25(OH)D testing performed at Calgary Laboratory Services in Alberta, Canada also found higher 25(OH)D levels in female than male patients and improved mean 25(OH)D levels later in adulthood (described as a nadir in early adulthood) [26]. A population-based study in São Paulo, Brazil of 636 participants showed similar correlations regarding age, sex, and mean 25(OH)D concentrations [27]. Other population studies, however, have shown higher 25(OH)D values in males than females [28]. Finally, seasonal/UV-B dependent variation in 25(OH)D levels have been well-characterized in numerous prior studies [29-35], as well as a more recent excellent report analyzing 3.44 million U.S. patient samples [36].

It is tempting to speculate that differences in 25(OH)D levels between females and males could also be due to differences in circulating vitamin D binding protein (DBP; also known as Gc globulin). For example, some previous studies have found higher circulating DBP levels in women versus men [37,38], including higher levels in pregnant women [9,38]. Interestingly, two prior studies have not shown a correlation between DBP levels and age, although their population sizes [n = 100 participants, men >45 years old and women >55 year old, ref [37]; n = 228 participants, age ranges 18–69, ref [38]] may theoretically preclude the ability to see any subtle trends if present. Furthermore, one of these studies actually demonstrated lower 25(OH)D levels in women versus men [37]. Until clinically-approved DBP assays are commercially available and/or DBP research assays are included in larger population studies, we may not have a complete picture of whether DBP levels vary throughout one’s lifetime. While the clinical relevance of circulating DBP levels to vitamin D status is not fully understood, studies have demonstrated that DBP polymorphisms can affect circulating 25(OH)D levels, as well as response to vitamin D supplementation and sun exposure [39-42]. Of particular relevance to the clinical laboratory, new evidence demonstrates an inverse correlation between the deviation of most vendor 25(OH)D immunoassay kits (versus an LC-MS/MS) and DBP levels, a finding that may explain some of the inaccuracies observed with 25(OH)D immunoassays [9].

One limitation of the present study is that patient ethnicity was not available in our datasets; therefore, results could not be subdivided by race. It is reasonable to assume that WCMC and UIHC 25(OH)D datasets may not be fully representative of national demographic patterns. U.S. Census data for 2010 reports an 86.1% white ethnicity in zip code 10065 (WCMC) and 88.8% white ethnicity in zip code 52242 (UIHC) [43]. These percentages, however, are likely much higher than the percent of white populations observed at these facilities, which both provide inpatient and outpatient care to patients far beyond a single zip code. No demographic information was available for the ARUP dataset, but it may be more representative of the U.S. population as a whole due to its status as a national reference laboratory. Finally, we were not able to control for any potential gender differences in the relative likelihood of visiting a healthcare provider in our patient populations.

Other limitations of the present report are the variability inherent to including multiple immunoassay platforms in our datasets (see Methods), as well as any potential short- and/or long-term changes in assay performance characteristics that can sometimes be observed in the clinical laboratory setting. While we observed differences in average 25(OH)D results across different assay platforms (see Table 2), it is important to note that ordering practices for 25(OH)D testing have dramatically shifted over time. A change from prior testing primarily for clinically suspected deficiency toward the more recent widespread utilization of such testing in otherwise healthy individuals likely confounds the attempt to utilize our dataset for direct assay comparisons. As our all of the testing, however, was ordered by clinicians and used in the management of patient care, we believe that it is appropriate to include all available data for patient averages and reference interval distributions in the present report, while acknowledging that some degree of bias may be introduced by including multiple assays in such analysis.

In our chart review, most cases of 25(OH)D levels greater than 150 ng/mL (potentially toxic) were due to vitamin D over-supplementation (especially use of 50,000 IU prescription vitamin D_2_ at weekly or greater intervals, intake of prescribed daily vitamin D that approximated or exceed weekly totals of >50,000 IU, and/or very heavy use of over-the-counter supplements outside of physician recommendations). Frequently, however, vitamin D intake was not well documented. In some cases, the dose recorded in the patient’s chart was far below what would be expected to cause potentially toxic levels. It is unclear whether this represented inaccurate/incomplete documentation or hypersensitivity to vitamin D.

It should be noted that elevated 25(OH)D results were uncommon at both UIHC and WCMC. For the UIHC population, only 27 of 60,237 tests (~0.05%) ordered over an approximately 13 year period had values >150 ng/mL. Of these 27 patients, only three had hypercalcemia and only one was hospitalized in critical condition. At WCMC, 15 of 57,433 orders for 25(OH)D by immunoassay (~0.03%) had values > 150 ng/mL. Of these 15, only four had hypercalcemia and only one was hospitalized (also in critical condition). While it was clear that documentation of over-the-counter supplementation was frequently absent or incomplete in our chart review, it is possible that vitamin D toxicity may only been suspected when symptoms (and/or laboratory findings) consistent with hypercalcemia were observed. In this era of increased attention to (and supplementation of) vitamin D, it would be prudent to keep the possibility of vitamin D toxicity in the differential diagnoses of patients receiving large doses of vitamin D_2_ or vitamin D_3_.

## Conclusions

In conclusion, the present report describes the order and result patterns for 25(OH)D testing at two large academic medical centers, and shows that these data are reflective of results observed at a national reference laboratory. Future studies on large populations may be necessary to fully understand vitamin D status throughout life.

## Competing interests

All authors (JRG, JTG, TCW, ER, and MDK) declare that they have no competing interests.

## Authors’ contributions

JRG and MDK were responsible for the design, acquisition, analysis, and interpretation of data. JTG provided statistical analysis in the generation of area plots. JRG, TCW, ER, and MDK conducted chart review. All authors (JRG, JTG, TCW, ER, and MDK) participated in drafting the manuscript and approve of the final submission.

## Authors’ information

JRG is Medical Director of the Automated Core Laboratory at ARUP Laboratories and Assistant Professor in the Department of Pathology at the University of Utah. JRG previously served as Assistant Director of the Core Laboratory at Weill Cornell Medical Center/New York Presbyterian Hospital. JTG is a visiting professor at Sacred Heart University and teaches undergraduate and graduate courses including research methods. TCW and ER are pathology residents in the Department of Pathology at the University of Iowa Hospitals and Clinics. MDK is Director of the Clinical Laboratories and Clinical Associate Professor in the Department of Pathology at University of Iowa Hospitals and Clinics.

## Pre-publication history

The pre-publication history for this paper can be accessed here:

http://www.biomedcentral.com/1472-6823/13/52/prepub
